# Detection and characterization of folded-chain clusters in the structured melt of isotactic polypropyl­ene

**DOI:** 10.1107/S2052252521003821

**Published:** 2021-05-15

**Authors:** Xiangyang Li, Jianjun Ding, Pujing Chen, Kang Zheng, Xian Zhang, Xingyou Tian

**Affiliations:** a Institute of Solid State Physics, CAS Key Laboratory of Photovoltaic and Energy Conservation Materials, Hefei Institutes of Physical Science, Chinese Academy of Sciences, Hefei, 230031, People’s Republic of China

**Keywords:** crystal engineering, crystal morphology, crystal-structure prediction, folded-chain clusters, lamellar systems, structured melts, isotactic polypropylene, SAXS

## Abstract

Despite ceaseless efforts in past decades, the memory effect of semi-crystalline polymers has not been elucidated completely yet. An important reason why is that residual lamellar crystals in the structured melt are difficult to characterize. Recently, we developed a new small-angle X-ray scattering (SAXS) theory [Li *et al.* (2019). *IUCrJ*, **6**, 968–983] and Fourier transform method [Li *et al.* (2020). *CrystEngComm*, **22**, 3042–3058] for lamellar crystals that could derive structural information from SAXS readily. In this study, we tried to employ the new theory and method to characterize residual lamellar crystals in the structured melt. It was found that although scattering peaks cannot be observed in raw scattering profiles, they actually exist. Subtracting free-melt scattering and multiplying by *q* ^4^ benefit the observation of these weak scattering peaks. With the new Fourier transform method, it was found that indeed as proposed previously, thicker lamellar crystals exist in the structured melt. To determine the lateral size of residual lamellar crystal especially, a new method was developed under the guidance of the new theory. With the new method, it was found that although the crystallinity is very low (∼1% at 174°C), the lateral sizes in the structured melts are still large, *e.g.* 45.3 nm at 174°C, much greater than the critical nucleation size. This implies that these residual lamellar crystals can act as athermal nuclei after quenching to a lower temperature, as proposed by Ziabicki & Alfonso [(1994). *Colloid Polym. Sci.* **272**, 1027–1042; (2002). *Macromol. Symp.* **185**, 211–231] more than 20 years ago. The methodologies proposed here could also be applied to other polymer lamellar systems.

## Abstract 2

A complete set of new methods have been proposed that can determine lamellar thickness, long period and lateral size of lamellar crystals readily and accurately.

## 1. Introduction   

As a unique phenomenon of polymer, memory effect has received increased attention over recent decades (Fillon *et al.*, 1993 ▸; Supaphol & Spruiell, 2000 ▸; Li *et al.*, 2013 ▸; Reid *et al.*, 2013 ▸). Initially, it referred to the fact that some special melts prepared slightly above the melting point could recover previous crystalline morphology after cooling to a lower temperature, seemingly having a memory on previous crystalline morphology (Fillon *et al.*, 1993 ▸). Later, it was extended to flow-induced crystallization. After shearing, if a melt was not quenched to a lower temperature immediately, the resulting morphology at the lower temperature, *i.e.* transcrystallinity, would disappear gradually with relaxation time, seeming as if the memory on previous shearing history was fading (Ziabicki & Alfonso, 2002 ▸; Azzurri & Alfonso, 2005 ▸). The former was named crystalline memory effect (Supaphol & Spruiell, 2000 ▸), while the latter was named flow memory effect (Azzurri & Alfonso, 2005 ▸).

Interestingly, many instruments, such as in differential scanning calorimetry (Lorenzo *et al.*, 2006 ▸), polarized optical microscopy (POM) (Li *et al.*, 2014 ▸) and nuclear magnetic resonance (Maus *et al.*, 2007 ▸), cannot detect any difference between free melt and the specially prepared melt. Evidently, in the specially prepared melt exist residual crystals. If no ordered structures survived in the melt, it would not have so many nuclei in the subsequent crystallization (Supaphol & Spruiell, 2000 ▸). This is why the specially prepared melt was also named structured melt (Zhang *et al.*, 2012 ▸; Maus *et al.*, 2007 ▸; Zhang & Martins, 2006 ▸) or memorized ordered melt (Ji *et al.*, 2016 ▸). Over recent decades, to elucidate the origin of memory effect, one has been seeking evidence for ordered structures (Sangroniz *et al.*, 2017 ▸; Martins *et al.*, 2010 ▸), especially the structural evidence from small-angle X-ray scattering (SAXS). Because if ordered structures do indeed exist, they are probably folded-chain clusters (Hong & Miyoshi, 2013 ▸, 2014 ▸; Hong *et al.*, 2015 ▸). Their thickness and lateral size are on a nanoscale level, while SAXS is the most powerful tool to detect such nanoscale structure.

Nevertheless, it is not an easy thing to obtain the above information with classical SAXS theory. Much like general scattering theory, classical SAXS theory assumes that scattering at a small wavevector is from all residual lamellar crystals. To obtain structural information, one must assume that the residual crystals have a fixed shape and determine all possible scatterings in different orientations, and then one can obtain structural parameters by fitting real scattering with theoretical scattering (Su *et al.*, 2008 ▸, 2009 ▸; Zhang *et al.*, 2012 ▸; Shen *et al.*, 2020 ▸). For example, Zhang *et al.* assumed that residual lamellar crystals have a cylindrical shape (Zhang *et al.*, 2012 ▸; Shen *et al.*, 2020 ▸). Scattering intensity from a cylinder is (Shen *et al.*, 2020 ▸)

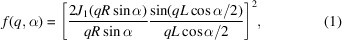

where *L* and *R* denote the height and radius of the cylinder, respectively, α is the angle between the scattering vector *q* and the cylinder axis, and *J*_1_(*x*) is the first-order Bessel function of the first kind.

Overall scattering is (Zhang *et al.*, 2012 ▸)

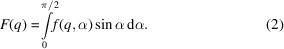

Fitting real scattering with equation (2), one could obtain *L* and *R*. However, equation (2) involves immense calculations. During fitting, the integration needs to be recalculated constantly until satisfactory parameters are obtained, which leads to fitting being a time-consuming process. Here, equation (2) does not consider size distribution. If considering the size distribution, it becomes more complicated.

**Figure 1:**
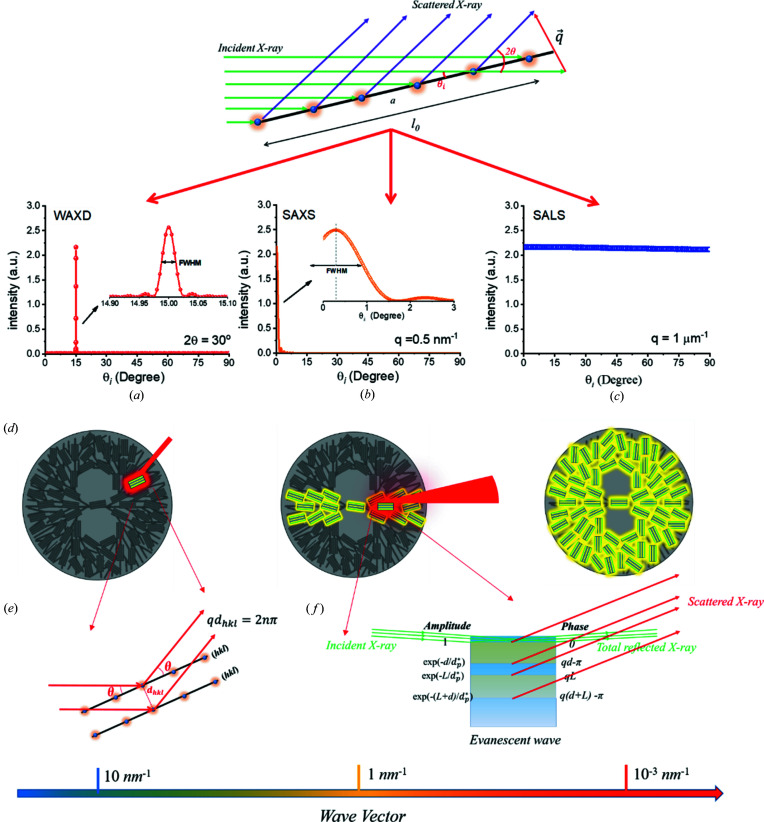
The influence of incident angle on the scattering intensity of a crystal plane at (*a*) 2θ = 30°, (*b*) *q* = 0.5 nm^−1^ and (*c*) *q* = 1 µm^−1^. Insets in (*a*) and (*b*) show the change of scattering intensity near θ_i_ = 15° and *q* = 0 nm^−1^, respectively. (*d*) Illustrations for lamellar crystals, which have significant contributions to scattering in WAXD, SAXS or SALS. (*e*) The diffraction mechanism in WAXD. (*f*) An illustration of the scattering of the interfacial electrons involved in the evanescent wave.

Recently, we investigated SAXS of polymer lamellar crystals again (Li *et al.*, 2019 ▸). It was found that SAXS is different from wide-angle X-ray scattering (WAXD). For a crystal plane with a lateral size of a few hundred nanometres, the typical size for real lamellar crystals, only when the incident angle (θ_i_) is strictly equal to the scattered angle (θ) can it form strong scattering in WAXD. While in SAXS, in a wide range near θ_i_ = θ, the crystal plane can form strong scattering due to small wavevector [see Figs. 1 ▸(*a*) and 1 ▸(*b*)]. However, it is also unlike small-angle light scattering (SALS). Assuming the wavelength employed in SALS is 632.8 nm, all orientations of crystal planes have strong scattering [see Fig. 1 ▸(*c*)], due to smaller wavevector (detail determination can be seen in Section S1 of the supporting information). If regarding the crystal plane as a filter, probably only a lamellar crystal in a spherulite has contributions to a diffraction in WAXD (Li *et al.*, 2019 ▸), a small fraction of lamellar crystals have contributions to a scattering in SAXS, while all of the lamellar crystals have contributions to a scattering in SALS, as sketched in Fig. 1 ▸(*d*).

More importantly, scattering mode changes significantly. In WAXD, the crystalline planes investigated normally are not parallel to the amorphous/crystalline interfaces within a lamellar stack, for example, orthorhombic (110) and (200) of polyethyl­ene (Wang *et al.*, 2002 ▸); monoclinic (110), (040) and (130) of isotactic polypropyl­ene (iPP) (Li *et al.*, 2017 ▸); and form I (110), (300) and (211) of poly(1-butene) (Su *et al.*, 2013 ▸). The scatterings from these crystal planes belong to asymmetric Bragg scattering. Under such mode, incident X-ray will not be reflected totally due to large intersection angle with the amorphous/crystalline interface. X-ray intensity can be regarded as invariant, since the adsorption of polymer for X-ray is very limited (de Jeu, 2016 ▸), and therefore it can employ kinematical diffraction theory to deal with WAXD. However, to obtain lamellar thickness and long period from SAXS, it must deal with the interference of the amorphous/crystalline interfaces. The scatterings from the amorphous/crystalline interfaces belong to symmetric Bragg scattering. Under such mode, total reflection of X-ray may occur (Als-Nielsen & McMorrow, 2011 ▸). When total reflection occurs, incident X-ray cannot enter the lamellar stack and induce electron scattering. Instead, an evanescent wave induced by incident X-ray will enter the lamellar stack and induce electron scattering. Nevertheless, due to rapid decay of the intensity of the evanescent wave, the scattering cannot be dealt with using kinematical diffraction theory. It should be dealt with using dynamical diffraction theory. Therefore, we built a new SAXS model of dynamical diffraction (Li *et al.*, 2019 ▸). As sketched in Fig. 1 ▸(*f*), when a beam of incident X-ray reaches a lamellar stack with a smaller intersection angle than the critical total reflection angle, it would induce an evanescent wave. The evanescent wave propagates downward and induces electron scattering. The scattering intensity decreases constantly with penetration depth because of the decay of the evanescent wave. Besides, a half-wave loss may occur at the interfaces from crystalline layer to amorphous layer, separating the scattering of interfacial electrons from that of bulk electrons. Calculations indicated that the scattering of interfacial electrons induced by the evanescent wave ( 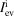 ) is the real origin of the SAXS signal of lamellar crystals (Li *et al.*, 2019 ▸). It is stronger than other scatterings, for example, the scattering induced directly by incident X-ray and the scattering of bulk electrons involved in total reflection ( 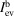 ).

Such a mechanism is unique in the SAXS of lamellar crystals. It will not take a dominant place in SALS and WAXD. The fact that the interfacial electrons involved in the evanescent wave can induce a strong scattering is mainly due to two points. Firstly, 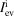 could be separated from 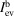 due to the difference in phase. As is well known, reflected light has a half-wave loss when light propagates from an optically denser medium to an optically thinner medium in normal incidence or grazing incidence (Born & Wolf, 1999 ▸). It also happens in X-ray; nevertheless, the amorphous phase is the optically denser medium, contrary to visible light (de Jeu, 2016 ▸). It does not occur in bulk. The difference separates 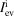 from 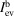 . Secondly, scattered X-rays from the interfaces exhibit significant difference in optical path. When the optical-path difference between adjacent interfaces is equal to a wavelength, it can induce strong scattering. For SALS, the wavelength employed normally is 632.8 nm. It is hard to induce such longer optical-path difference between adjacent interfaces because their distance normally is only 10 nm. Scatterings from interfaces in SALS are probably canceled out. Generally, the characteristic penetration depth of the evanescent wave 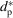 is a few wavelengths (Born & Wolf, 1999 ▸). For SALS, 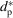 is in the microscale. Scattering intensities at every interface of lamellar stack are almost equal. Due to equal intensity and half-wave loss, 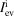 is highly canceled out (an estimation for 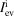 in SALS can be seen in Section S2). As for WAXD, no half-wave loss exists because of the absence of total reflection. Therefore, for lamellar crystals, SAXS is neither the same as SALS nor the same as WAXD. We cannot employ the methods in WAXD or SALS to deal with SAXS of lamellar crystals. Equations (1) and (2) adopt the SALS method to deal with SAXS of lamellar crystals, which could be inappropriate.

Nevertheless, SAXS is closer to WAXD in some sense. For SAXS and WAXD, most of the lamellar crystals will not form strong scattering. Only quite a few lamellar crystals can form strong scattering. Nevertheless, in WAXD, the lamellar crystals that can form strong scattering are those satisfying the Bragg condition; while in SAXS, the lamellar crystals that can form strong scattering are those satisfying the total reflection condition. The narrow full width at half-maximum (FWHM) in WAXD is like a high-effective filter, excluding most of the lamellar crystals. While in SAXS, the total reflection condition replaces the role of the FWHM. As reported earlier (Li *et al.*, 2019 ▸), the critical total reflection angle at the amorphous/crystalline interface of iPP is ∼0.034°, close to the FWHM in the wide-angle range, for example the FWHM is 0.029° at 2θ = 30° in the inset of Fig. 1 ▸(*a*).

Based on the new scattering theory, we wanted to investigate residual lamellar crystals in the memorized ordered melt. Assuming that these residual lamellar crystals still adopt a folded-chain conformation, the only difference from lamellar crystals is that they have a smaller lateral size, and therefore the new scattering theory is still applicable. Lower-temperature crystallized isotactic polypropyl­ene (LM-iPP) was employed as a model system since we are familiar with the system. With POM and Fourier transform infrared spectroscopy, we found that although a spherulite had disappeared completely in the temperature window of 170–174°C, it still can recover after cooling to a lower temperature, indicating the memory on previous crystalline morphology (Li *et al.*, 2014 ▸).

## 2. Experimental section   

### 2.1. Materials   

Raw iPP material (trade name: PP531Ph) employed in the study has a melt flow index of ∼0.3 g per 10 min (230°C per 2.16 kg, ASTM D1505), a weight-average molecular weight of 720 kg mol^−1^,and a polydispersity index of 4.8, which was kindly supplied by SABIC Europe. The iPP films used for the memory-effect experiments were prepared by compression molding, after removal of thermal history at 220°C for 5 min. The thickness is ∼200 µm.

### 2.2. POM   

To confirm the memory effect of LM-iPP, an Olympus POM was employed, which was equipped with a charge-coupled device and a heating device. The spherulites used for the memory-effect experiments were prepared *in situ*. After removing thermal history at 220°C for 5 min, the iPP melt was quenched to 135°C and kept until a suitable spherulite grew to ∼200 µm. Afterwards, the temperature was increased quickly to a higher temperature (170, 172 and 174°C) and kept for 5 min to melt the spherulite. After melting, it was quenched to 135°C again for recrystallization. During recrystallization, a POM was taken automatically every 10 s with self-contained software.

### 2.3. Synchrotron SAXS   

To investigate ordered structures in the memorized ordered melt, synchrotron SAXS at beamline BL16B of the Shanghai Synchrotron Radiation Facility (SSRF) was employed. The LM-iPP sample used for SAXS measurements was prepared by isothermal crystallization at 135°C after removal of thermal history at 220°C for 5 min.

Two-dimensional SAXS patterns were collected during heating of the iPP sample from 135 to 220°C with a Mar165 CCD equipped in the synchrotron SAXS. The heating rate was 4°C min^−1^, controlled by a Linkam hot stage at the beamline. The wavelength of the X-ray beam was fixed at 0.124 nm. The sample-to-detector distance was set to 2500 mm. During heating, a SAXS pattern was collected every half a minute. The exposure time was 20 s. After collection, 2D SAXS patterns were transited to 1D scattering profiles with the help of *FIT2D* software from the European Synchrotron Radiation Facility (Hammersley, 2016 ▸).

## 3. Results and discussion   

### 3.1. POM   

**Figure 2:**
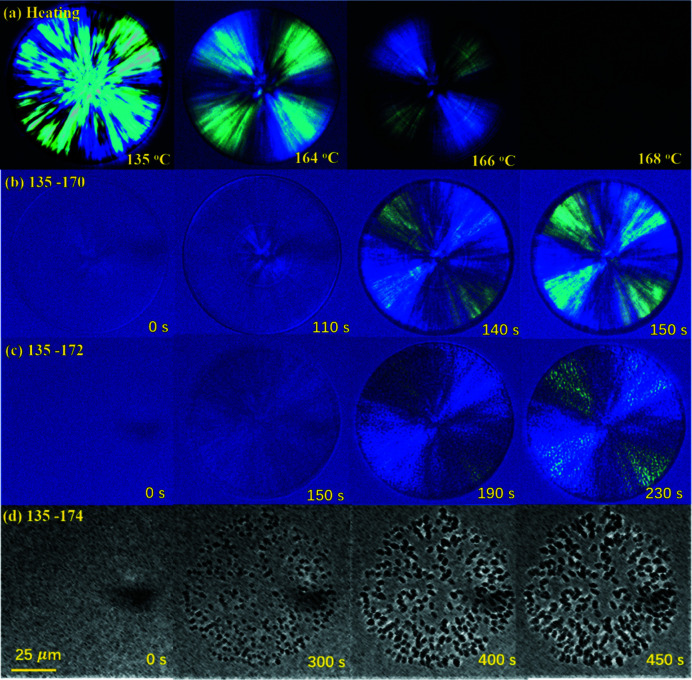
(*a*) Morphological change during heating. The recover processes of spherulites at 135°C after melting at (*b*) 170, (*c*) 172 or (*d*) 174°C. To see the crystal grains in (*c*) clearly, colored images were changed to gray images.

Before investigating ordered structures in the structured melt, we checked the memory effect of LM-iPP again, using POM equipped with a primary red filter (λ plate) and a heating device. As found previously (Li *et al.*, 2003 ▸, 2014 ▸), the spherulite formed at 135°C disappeared above 170°C [see Fig. 2 ▸(*a*)]. Nevertheless, if the melting temperature was not higher than 174°C, the disappeared spherulite would appear again after cooling to 135°C, as seen in Figs. 2 ▸(*b*)–2 ▸(*d*). Besides, negative spherulite changed to positive spherulite after recrystallization, as reported by Li *et al.* (2003 ▸). Using scanning electron microscopy, Li *et al.* (2006 ▸) found that the spherulite growing from the free melt was mainly composed of radial lamellae, while the spherulite recovered from the structured melt was composed of radial and tangential lamellae of equal number, which could be the reason that the optical character changed after recrystallization.

### 3.2. SAXS   

#### 3.2.1. Raw SAXS   

**Figure 3:**
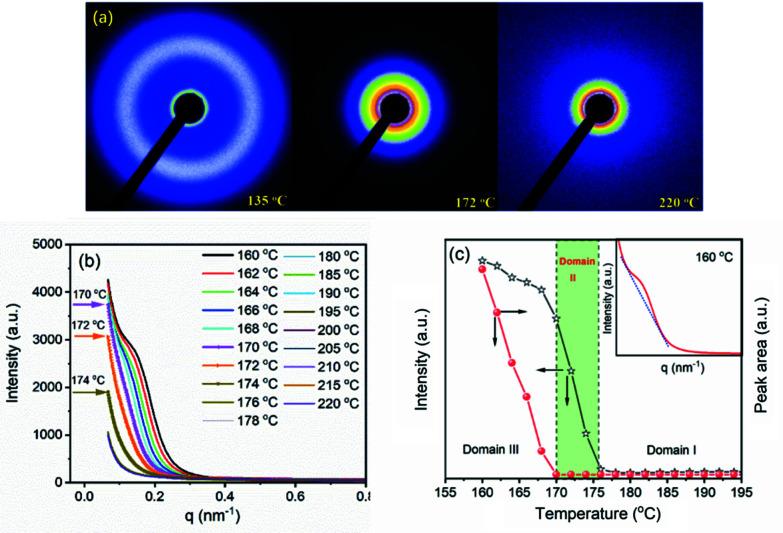
(*a*) Selected 2D SAXS patterns collected during heating. (*b*) One-dimensional SAXS profiles from 160 to 220°C. (*c*) Changes in peak intensity and area at *q* = 0.14 nm^−1^ during heating. The inset displays the method to determine peak area. To exclude the influence of scattering intensity on the peak area, a tangent line was drawn below the scattering peak, and only the area between the scattering peak and the tangent line was determined.

First, let us see what raw scatterings look like. Fig. 3 ▸(*a*) shows selected 2D SAXS patterns obtained during heating from 135 to 220°C. At 135°C, there exists a clear scattering ring, indicating the existence of lamellar stacks. The scattering at 172°C is similar to that at 220°C, no scattering ring can be seen. As mentioned above, the melt at 172°C is the memorized ordered melt, while the melt at 220°C is the free melt. Directly from the 2D scattering patterns, it is hard to infer what difference exists in structure between the memorized ordered melt and the free melt. To compare these scattering patterns more meticulously, 2D SAXS patterns were transited to 1D scattering profiles with the help of *FIT2D* software. Fig. 3 ▸(*b*) shows scattering profiles above 160°C. At 160°C, the scattering peak becomes weak, indicating that melting has begun. As the temperature increases, the scattering peak becomes weaker and weaker. At 170°C, the scattering peak disappears. Nevertheless, it still has a high intensity. The intensity decreases with increasing temperature further until 176°C. From 176°C on, the scattering intensity does not decrease any more. Scattering profiles above 176°C almost overlap completely. With the changes in peak area and intensity, we can divide the temperature into three domains, as shown in Fig. 3 ▸(*c*). In Domain III, *i.e.* the temperature is smaller than 170°C, both peak area and intensity decrease with temperature. In Domain II (170 ≤ *T* < 176°C) the peak area becomes zero. It does not change with temperature. Nevertheless, the peak intensity still decreases with temperature. In Domain I (*T* ≥ 176°C) both peak area and peak intensity reach the minimum values. They do not change with temperature. The melts in Domain II are so-called structured melts. The higher intensity in Domain II implies the existence of some ordered structures. Nevertheless, no more information can be obtained from the raw scatterings directly.

#### 3.2.2. Determinations of long period and lamellar thickness   

Let us determine long period and lamellar thickness first with the new SAXS theory proposed by us. Unlike classical SAXS theory, the new theory assumes that the SAXS signal is only from interfacial electrons in a few lamellar stacks, which satisfys the total reflection condition. Other lamellar stacks have negligible contributions to the SAXS signal because of weak form factor, weak structure factor and/or destructive interference (Li *et al.*, 2019 ▸). Therefore, we should obtain structural information from the scattering of the interfacial electrons induced by the evanescent wave 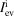 .

Here exist two questions. First, how to obtain 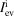 from real scattering? Second, how to obtain lamellar thickness and long period from 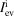 ? The first question is easily solved. Since other lamellar stacks have no contribution to the SAXS signal, we can assume that their scattering is equal to a scattering of a free melt with the same irradiated volume. Under such assumption, 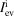 can be estimated by the following equation:

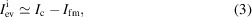

where *I*_c_ is a scattering of a crystallized polymer, while *I*_fm_ is a scattering of a free melt with the same irradiated volume as the crystallized polymer. For quiescent crystallization, *I*_fm_ and *I*_c_ could be replaced by the scatterings before and after crystallization, respectively, because the thickness of the sample in the sample chamber normally remains unchanged. Similarly, they could be also replaced by the scatterings before and after melting, if the thickness remains unchanged.

To solve the second question, let us look at the scattering equation of 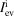 , which was given in the last study (Li *et al.*, 2019 ▸):

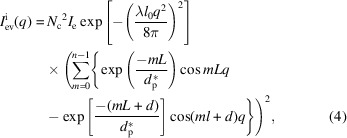

where *N*_c_ is the electron number on the (00*l*) crystal plane, *I*_e_ is the scattering intensity of a single electron, *l*_0_ is the lateral size of the lamellar crystal, λ is the wavelength of X-ray, *d* is the lamellar thickness, *L* is the long period, *n* is the number of lamellar crystals in the lamellar stack and 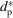 is the characteristic penetration depth of the evanescent wave. It can be divided into two parts:

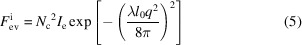

and

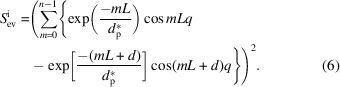

The former part 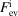 represents the scattering intensity of the first interface, which can be regarded as the form factor. It affects the overall scattering intensity. The latter part 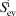 represents the interference intensity of all interfaces, which can be regarded as the structure factor. The lateral size only exists in the form factor. With melting, the lateral size reduces constantly. The scattering intensity also decreases, since *N*_c_ decreases with the lateral size. Long period and lamellar thickness only exist in the structure factor. They dominate scattering peak positions. Therefore, lateral size should be obtained from the form factor, while the long period and lamellar thickness should be obtained from the structure factor.

Nevertheless, in most cases, the form factor and the structure factor are hardly separated from scattering intensity. In another study of ours (Li *et al.*, 2020 ▸), we explored the method to obtain lamellar thickness and long period from 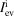 . It was found that multiplying by *q* ^4^ can reduce the influence of form factor effectively. Besides, multiplying by *q* ^4^ could also reduce the scattering of the bulk electrons induced by the evanescent wave in a lamellar stack 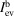 (detailed discussion can be found in Section S3). After multiplying by *q* ^4^, the overall scattering induced by the evanescent wave is controlled by 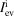 [see Fig. S4(*c*) of the supporting information]. After reducing the influence of form factor, lamellar thickness and long period can be obtained readily from Fourier transform,

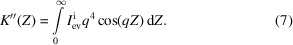

The function obtained, 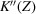 , is the so-called interface distribution function (IDF). Combining equations (3) and (7), the lamellar stack and long period can be obtained from real scattering,

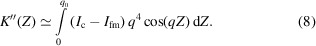

Here *q*_0_ is the wavevector at the intersection point of the scattering profiles before and after crystallization. This only employs the scattering data before *q*_0_ to obtain lamellar thickness and long period, which can effectively reduce the influence of Porod scattering (Li *et al.*, 2020 ▸).

**Figure 4:**
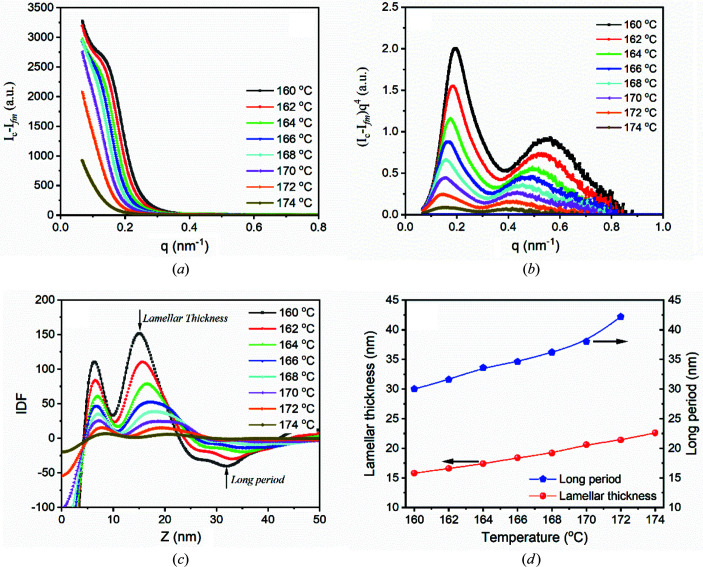
(*a*) Scattering profiles after subtracting the scattering at 220°C. (*b*) (*I*_c_ − *I*_fm_)*q* ^4^ and (*c*) IDFs in the range of 160–174°C. (*d*) Long periods and lamellar thicknesses obtained from (*c*).

With the new method, let us see if it is possible to obtain lamellar thickness and long period from the scatterings of structured melts. Fig. 4 ▸(*a*) shows scattering profiles after subtracting the scattering at 220°C, which can be regarded as *I*_fm_. No scattering peaks can still be seen from the scattering profiles. This could be due to the influence of 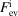 and 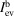 . To reduce their influence, scattering profiles were corrected further by multiplying by *q* ^4^. Fig. 4 ▸(*b*) shows (*I*_c_ − *I*_fm_)*q* ^4^ from 160 to 174°C. It was found that the long-period peak actually exists in the range of 170–174°C but its intensity becomes weak. With increasing temperature, the long-period peak shifts slightly to the small *q* range, indicating the increase of long period. There also exists another peak, located in the range of 0.3–0.6 nm^−1^. This weak peak is barely reported because it is too weak to be observed in the raw scattering. Only after reducing the influence of form factor can it be observed. It is not the second-order peak of the long-period peak, since its position is not equal to two times that of the long-period peak. In the last study (Li *et al.*, 2019 ▸), we found that it might be from interference of adjacent interfaces in a lamellar crystal, therefore we called it the lamellar peak. As well as the long-period peak, the lamellar peak shifts to the small *q* range, implying lamellar thickening during heating. To observe the changes of lamellar thickness and long period directly, IDFs at various temperatures were determined with equation (8). Fig. 4 ▸(*c*) displays the IDFs that were determined. From Fig. 4 ▸(*c*), it can be found that the lamellar thickness increases from 15.8 nm at 160°C to 21.4 nm at 172°C. The lamellar peak at 174°C is very weak, locating around 22.6 nm. The long period increases from 30 nm at 160°C to 42.2 nm at 172°C, as shown in Fig. 4 ▸(*d*). The long-period peak at 174°C is too weak to be identified.

#### 3.2.3. Determination of lateral size   

Now, let us look at how to obtain lateral size from the form factor. Before exploring the method to determine the lateral size from the form factor, we would like to revise equation (5) slightly. The electron number *N*_c_ in the equation is changed to *l*_0_/*a*, where *a* is the distance between adjacent electrons and *l*_0_ is the lateral size, as mentioned above. This change benefits observing the influence of lateral size on the form factor thoroughly. After the change, equation (5) becomes

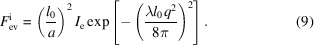

**Figure 5:**
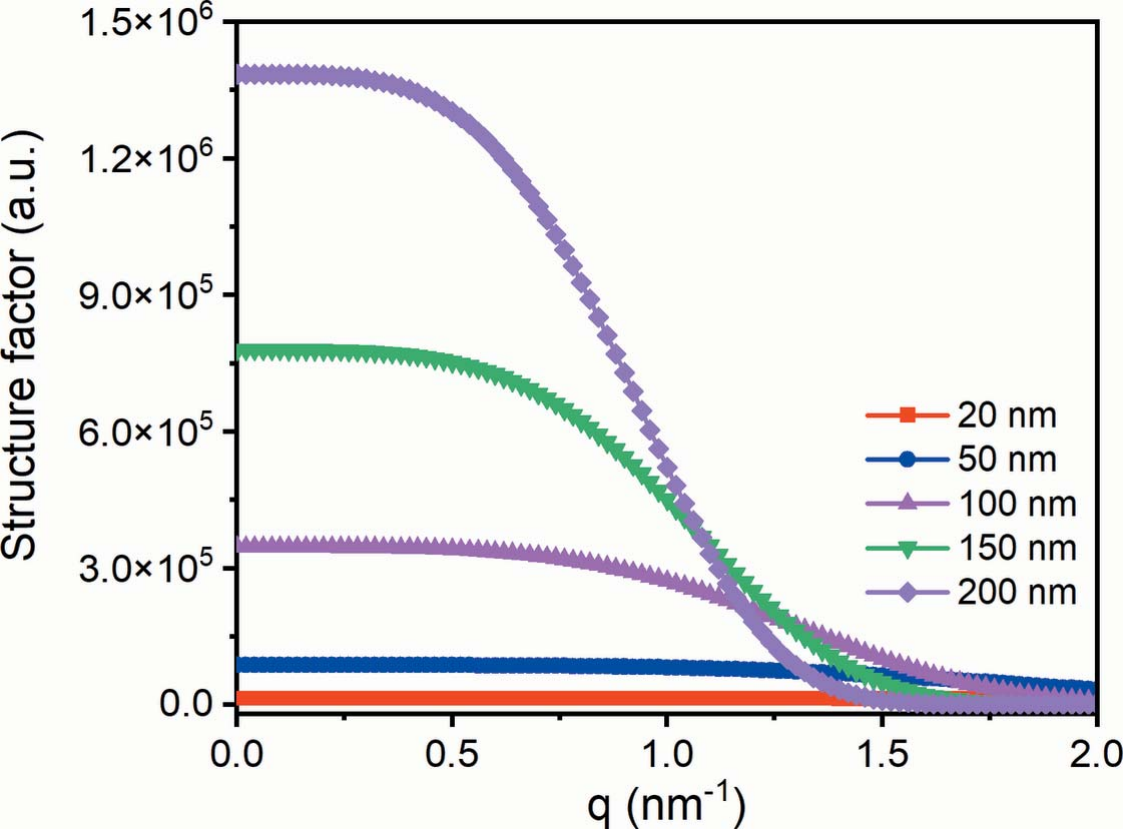
The change in form factor with the decrease in lateral size.

With the revised equation, let us simulate how the form factor changes with lateral size during melting. Fig. 5 ▸ displays form factors at various lateral sizes, assuming *a* = 0.17 nm, *I*_e_ = 1 and λ = 0.124 nm. From Fig. 5 ▸, it can be found that arbitrarily a form factor can be divided into two regions, small *q* region A and high *q* region B. In region A, form factor remains basically unchanged.

In region B, the form factor decreases monotonously. From equation (9), it is easily determined that the plateau value in region A is proportional to the square of the lateral size, while the slope in region B is related to lateral size. The greater the lateral size, the steeper the slope. Therefore, there are two methods to determine the lateral size.

The first method is from the slope. From equation (9), it is easily determined that the decrease results from the exponential decay function in the form factor. If we take a logarithm of equation (5), 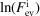 is proportional to *q* ^4^:

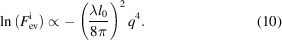

Its slope is only determined by the lateral size. Therefore, we can obtain the lateral size from the slope directly,

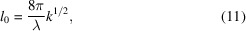

where *k* is the slope.

The second method is from the plateau value. From equation (9), it can be seen that if a plateau value and the lateral size corresponding to the plateau value are known, we can obtain lateral size corresponding to another plateau value,

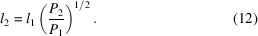

Here *P*_1_ and *P*_2_ are plateau values corresponding to lateral sizes of *l*_1_ and *l*_2_, respectively.

The first method is a direct method, which only needs the slope *k*. The second method is an indirect method, which needs at least a lateral size corresponding to a plateau value. Nevertheless, these two methods face the same problem, which is, how to avoid the influence of the structure factor 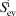 . Taking the logarithm of equation (4), we can obtain

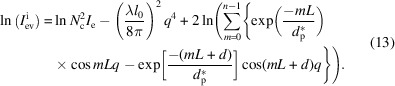

This is composed of three terms. The first term is a constant, having no relation with the wavevector *q*. The second term is proportional to *q* ^4^. The third term is a natural logarithm function, which also contains many terms. Every term in the natural logarithm function is composed of an exponential decay function and a cosine function. The first three terms or said three maximum terms are 1, 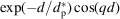 and 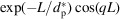 . Since the exponential decay functions are much smaller than 1, equation (13) can be reduced to

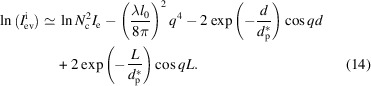

The first two terms are from the form factor, while the latter two terms are from the structure factor. When *q* > 1, the second term is much greater than the third and fourth terms. Then, equation (14) can be reduced further to

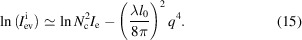

Therefore, if we plotted 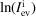 against *q* ^4^, the slope would be determined only by the form factor, or more exactly, the lateral size. Conversely, the lateral size could be estimated from the slope in the high *q* range.

To fully utilize the scattering in the high *q* range to determine the lateral size, we made a slight correction for the scattering. In equation (3), overall scattering before crystallization *I*_fm_ was subtracted. The subtraction is actually a bit more than what should be subtracted. It should only subtract the scattering of the melt that will not transform to lamellar stacks satisfying the total reflection condition. Assuming that in the erroneously subtracted melt some folded-chain clusters satisfying the total reflection condition exist, equation (3) actually is the difference in scattering intensity between a folded-chain embryo and a lamellar crystal,

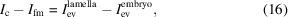

where 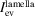 and 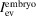 are the scatterings of a lamellar crystal and an embryo induced by a evanescent wave, respectively. As seen in Fig. 5 ▸, although the embryo has weak scattering, its scattering intensity can remain unchanged in a wide *q* range. While for lamellar crystal, despite having a strong scattering, its scattering intensity decreases rapidly. These two scattering profiles will inevitably intersect at a high wavevector, which is, *q*_0_ in equation (9). Actually, if not subtracting 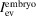 , scatterings before and after crystallization should be close infinitely in the high *q* range since the scattering induced by the evanescent wave does not play a leading role in higher *q* range anymore. Therefore, *I*_fm_ was corrected slightly,

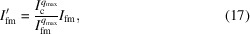

where 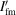 represents the scattering of the melt that will not transform to the lamellar stack satisfying the total reflection condition, while 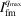 and 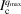 are scattering intensities before and after crystallization at the maximum measurable wavevector in the SAXS measurement, respectively. Accordingly, equation (3) can be revised slightly as

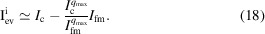

Combining equation (15) and (18), it could estimate the lateral size with the scattering in the high *q* range.

**Figure 6:**
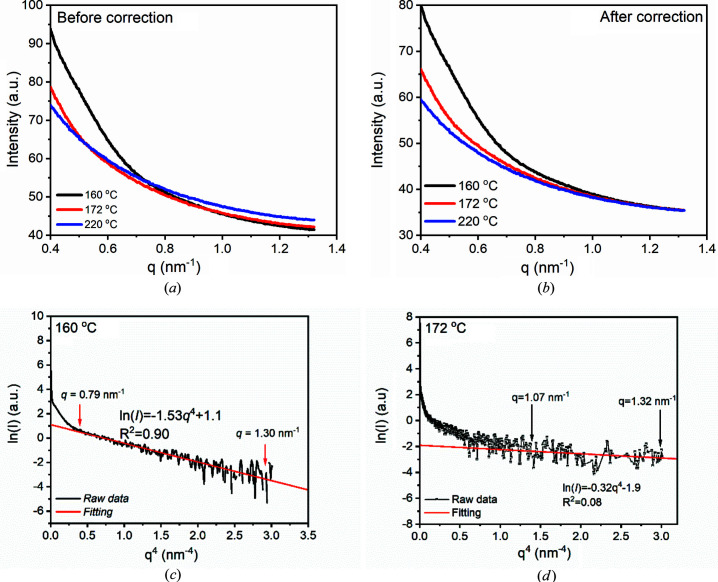
Scatterings at 160, 172 and 220°C, (*a*) before and (*b*) after correction. Plots of ln(*I*) and *q* ^4^ at (*c*) 160 and (*d*) 172°C.

Now, let us see whether the direct method is feasible, with scatterings at 160, 172 and 220°C as examples. Figs. 6 ▸(*a*) and 6 ▸(*b*) show scatterings at 160, 172 and 220°C before and after correction. Before correction, scattering profiles at 160 and 172°C intersect with the scattering profile at 220°C at 0.53 and 0.75 nm^−1^, respectively. The maximum measurable wavevector is ∼1.32 nm^−1^ due to the limitation of the detector. After the correction, scattering profiles at 160 and 220°C overlap from 0.8 nm^−1^, while scattering profiles at 172 and 220°C overlap only after ∼1.0 nm^−1^. The difference in scattering intensity can be regarded as 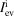 . Evidently, 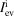 at 160°C is greater than that at 172°C.

With 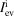 , lateral sizes at various temperatures were estimated using equation (15). Fig. 6 ▸(*c*) displays the plot of 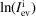 against *q* ^4^ at 160°C. Clearly, there exists a linear region, ranging from 0.79 to 1.30 nm^−1^. Through linear fitting, it is found that the slope is ∼1.53. With equation (11), it can be found that the lateral size is ∼251 nm. We also estimated the lateral size at 172°C. It seems that a linear region also exists, ranging from 1.07 to 1.32 nm^−1^. Its slope is ∼0.32. Estimating with equation (11), the lateral size is ∼115 nm. Nevertheless, it is hard to ensure the reliability of the lateral size. A great noise exists because of negligible difference between the scattering profiles at 172 and 220°C. The great noise leads to a small coefficient of determination *R* ^2^, which is only 0.08. With such a small *R* ^2^, the reliability of the lateral size cannot be ensured. Evidently, the first method is not suitable for determining lateral size of a small crystal grain or embryo.

The direct method fails to obtain the lateral size at high melting temperature. Let us see if the indirect method can be employed to obtain the lateral size or not. However, no plateau can be observed in real SAXS. This is probably due to the influence of 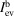 . As seen in Fig. S4(*b*), in the small *q* range, 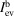 is much greater than 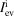 . Its intensity is inversely proportional to *q* ^4^. With the increase of *q*, it decreases rapidly, which will cover the plateau in 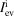 . Structure factor 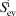 also has an influence on the plateau. As mentioned above, structure factor and form factor are hard to separate in practice. Due to the existence of structure factor, the plateau in the form factor is difficult to observe.

Although the indirect method cannot be employed to determine lateral size, we can obtain some implications from it. Equation (12) actually indicates that the ratio of lateral size is equal to the square of the ratio of the scattering peak intensities/area. This can also be understood as the crystal volume is proportional to the scattering peak area, since equation (12) can also be written as

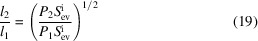

or

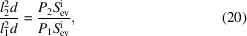

if assuming that the lamellar thickness and long period remained unchanged during melting. Here *d* is lamellar thickness and 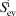 is structure factor. With this understanding, Equation (20) can be extended to

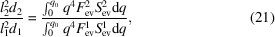

where *d*_1_ and *d*_2_ are lamellar thicknesses at lateral sizes of *l*_1_ and *l*_2_, respectively. Furthermore, 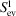 and 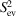 are structure factors at lateral sizes of *l*_1_ and *l*_2_, respectively. The plateau values *P*_1_ and *P*_2_ in equation (20) are replaced by 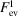 and 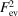 , respectively. To reduce the influence of 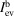 , the scattering was corrected by multiplying by *q* ^4^. As seen in Fig. S4(*c*), multiplying by *q* ^4^ can reduce 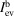 effectively. After multiplying by *q* ^4^, 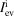 will be dominated in the overall small *q* range. Since the denominator and numerator in the right side of equation (21) represent peak areas in the plots of *Iq* ^4^ at the lateral sizes of *l*_1_ and *l*_2_, respectively, equation (21) can also be written as

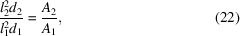

where *A*_1_ and *A*_2_ are scattering peak areas in the plots of *Iq* ^4^ at the lateral sizes of *l*_1_ and *l*_2_, respectively.

This is different from WAXD. In WAXD, peak area is directly utilized in the plot of scattering intensity to represent the amount of crystals. This is easily understood. In the wide-angle range, the Bragg condition is strictly satisfied. Only when the incident angle (θ_i_) is strictly equal to the scattering angle (θ) can strong scattering form. The form factor can be written as

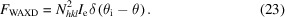

Here *N*_*hkl*_ is the electron number on a crystal plane. The intensity of the form factor has nothing to do with the wavevector. However, in SAXS, even when θ_i_ is not equal to θ, significant scattering can form due to small wavevector, which results in the exponential decay function. We need to remove the influence of the exponential decay function. Multiplying by *q* ^4^ can eliminate such influence, as discussed in the last study (Li *et al.*, 2019 ▸). This is a significant difference between SAXS and WAXD. With equation (22), we can estimate lateral size with the following equation:

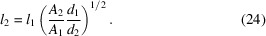

With the new approach, let us see if the lateral sizes at high melting temperatures can be determined or not. From equation (24), to determine lateral size corresponding to a particular scattering peak, one needs to know its lamellar thicknesses and scattering peak area and the lamellar thicknesses and area of a scattering peak with known lateral size. The lamellar thicknesses at various temperatures have been obtained. Therefore, we just need to determine scattering peak area at various temperatures.

**Figure 7:**
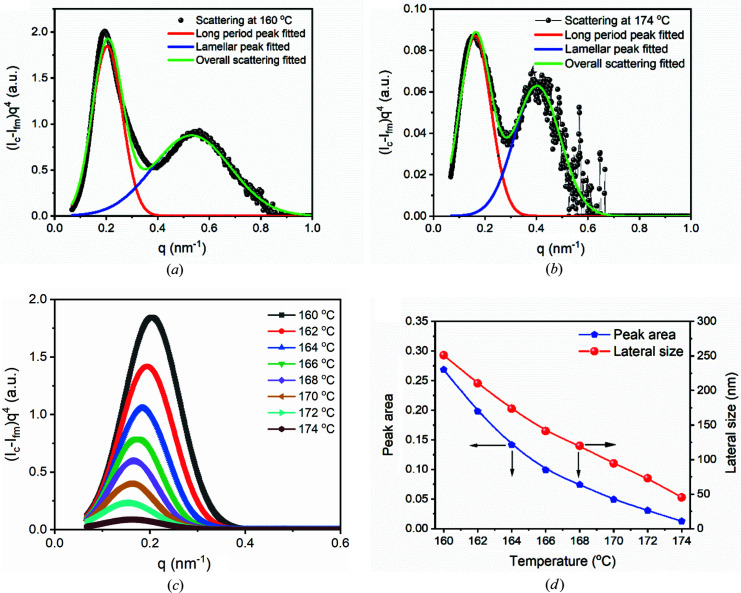
Multi-peak fitting for *Iq* ^4^ at (*a*) 160 and (*b*) 174°C. (*c*) First peaks obtained at temperatures between 160 and 174°C through fitting. (*d*) Changes in peak area and lateral size during heating.

Here, long-period peaks were chosen because of their higher intensities. To separate long-period peaks and lamellar peaks in the plots of (*I*_c_ − *I*_fm_)*q* ^4^, a multi-peak fitting procedure contained in the *Origin* software was employed (OriginLab, Northampton, Massachusetts, USA). Figs. 7 ▸(*a*) and 7 ▸(*b*) display fitting processes at 160 and 174°C. From Figs. 7 ▸(*a*) and 7 ▸(*b*), it can be seen that the long-period peak and the lamellar peak can be well separated. Fig. 7 ▸(*c*) shows the long-period peaks obtained through fitting in the range of 160–174°C. We determined the peak area and plotted it in Fig. 7 ▸(*d*). The peak area decreases with the increase of temperature. With the peak area and lamellar thicknesses determined, we estimated the lateral size using equation (24). The determined lateral sizes are also plotted in Fig. 7 ▸(*d*). The lateral sizes at 170, 172 and 174°C are 94.3, 72.9 and 45.3 nm, respectively. All of these values are greater than the critical nucleation size reported for iPP. Employing nanoporous alumina, Duran *et al.* (2011 ▸) found that the critical nucleation size of iPP was ∼20 nm. The larger lateral sizes at 170, 172 and 174°C mean that after quenching to a lower temperature, the folded-chain clusters in the memorized ordered melt can act as athermal nuclei. Therefore, it does not need to overcome the nucleation barrier for further growth. Twenty-six years ago, Ziabicki & Alfonso (1994 ▸) conjectured that athermal nuclei existed in the memorized ordered melt. However, it has not been demonstrated because of the difficulty to determine the lateral size. With the new SAXS theory, we have demonstrated this assumption.

Besides, from Fig. 7 ▸(*d*), it can be seen that the lateral size decreases linearly with temperature. On average, the lateral size decreases by 29.4 nm every 2°C during heating from 160 to 174°C at a rate of 4°C min^−1^. Assuming that the melting rate remains unchanged, the lateral size at 176°C will be 15.9 nm, lower than the critical nucleation size reported by Duran *et al.* (2011 ▸). This predicts that the clusters at 176°C will hardly nucleate directly when quenching to a lower temperature. This prediction is in agreement with the POM observation (Li *et al.*, 2014 ▸).

#### 3.2.4. Estimating crystallinity in the memorized ordered melt   

It seems incredible that in the memorized ordered melts there are still such larger lateral sizes. The larger lateral sizes seemingly imply the existence of a great number of crystals. While at such a high temperature, the crystallinity had been extremely low. To check it, we estimated the crystallinity at 174°C.

As well as WAXD, SAXS can also be used to estimate the crystallinity. Nevertheless, unlike WAXD, it cannot be applied to partial-melting or crystallizing samples. This is because it employs the ratio of lamellar thickness to long period to estimate the crystallinity,

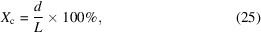

which does not consider the amorphous component outside lamellar stacks. If employing this method to estimate crystallinity in a partial-melting or crystallizing polymer, it would lead to a high estimation for crystallinity. To determine crystallinity in a partial-melting polymer, it would be better to first determine the crystallinity in a completely crystallized sample and then estimate crystallinity in a partial-melting polymer by comparing scattering peak area before and after melting,

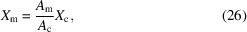

where *A*_c_ and *A*_m_ are scattering peak area before and after melting, respectively.

**Figure 8:**
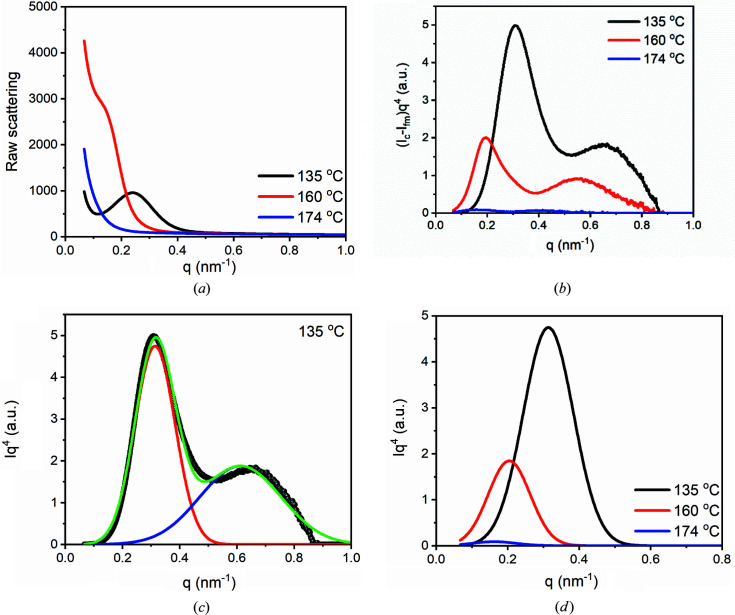
(*a*) Raw scattering at 135, 160 and 174°C. (*b*) The plots of *Iq* ^4^ at 135, 160 and 174°C. (*c*) Multi-peak fitting for *Iq* ^4^ at 135°C. (*d*) Long-period peaks at 135, 160 and 174°C.

To estimate the crystallinity at 174°C, SAXS at 135°C was taken as the reference. The crystallinity at 160°C was also determined as a comparison. Fig. 8 ▸(*a*) shows raw scattering profiles at 135, 160 and 174°C. From Fig. 8 ▸(*a*), it can be seen that the scattering at 135°C has a strong scattering peak. The scattering peak becomes weak at 160°C. While in the scattering at 174°C, it is hard to observe the scattering peak. We determined the peak area at 135 and 160°C, with the method in the inset of Fig. 3 ▸(*c*). The peak area at 135°C is ∼3.06 times that at 160°C, implying that about two thirds of crystals have been melted.

As discussed above, we should utilize scattering peaks in the plot of (*I*_c_ − *I*_fm_)*q* ^4^ to evaluate crystallinity. Fig. 8 ▸(*b*) shows (*I*_c_ − *I*_fm_)*q* ^4^ at 135, 160 and 174°C. From Fig. 8 ▸(*b*), it can be seen that scattering peaks at 160°C are much weaker than those at 135°C. The scattering at 174°C actually also contains scattering peaks, which are shown in Fig. 7 ▸(*b*). The peaks at 174°C are weaker than those at 135 and 160°C.

To compare scattering peak areas more accurately, we separated the scattering peaks in the (*I*_c_ − *I*_fm_)*q* ^4^ and only utilized a single peak area to evaluate the change of crystallinity. Fig. 8 ▸(*c*) shows the fitting process for the scattering at 135°C. The scatterings at 160 and 174°C are shown in Figs. 7 ▸(*a*) and 7 ▸(*b*). Fig. 8 ▸(*d*) displays long-period peaks fitting at the three temperatures. We determined the scattering peak area. The scattering peak area at 135°C is ∼3.12 times that at 160°C, close to the estimation with the peak area in the raw scattering. The peak areas at 135 and 160°C are ∼66.8 and 21.4 times that at 174°C, respectively.

**Figure 9:**
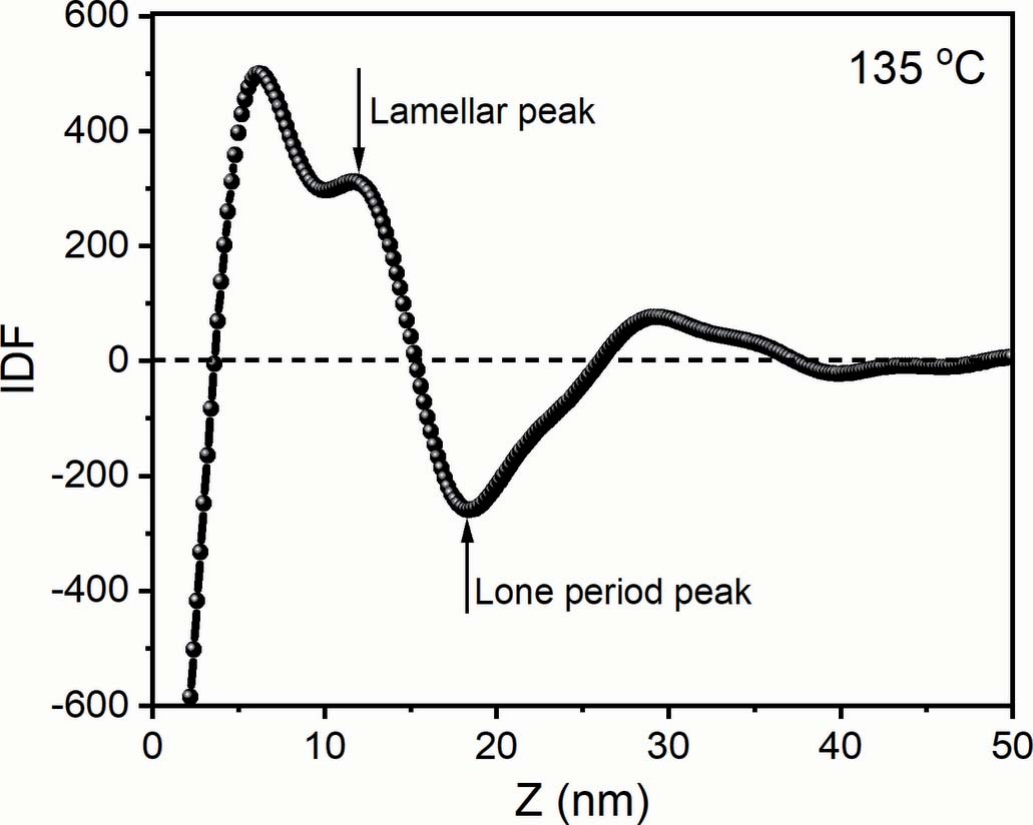
The IDF at 135°C

We determined the IDF at 135°C, as shown in Fig. 9 ▸. The lamellar thickness and long period are 11.8 nm and 18.2 nm, respectively. According to equation (25), the crystallinity is ∼64.8%. From this value and the ratios of peak area obtained above, we estimated the crystallinities at 160 and 174°C using equation (26). The crystallinities at 160 and 174°C are ∼20.8 and 0.97%. The crystallinity at 174°C is lower than 1%, close to or lower than the detection limitation of many instruments (Wang *et al.*, 2000 ▸). This could be the reason why many instruments cannot detect crystals in the memorized ordered melt.

## 4. Conclusions   

To summarize, by employing the new SAXS theory, we successfully determined folded-chain structures in a memorized melt. It was found that although the crystallinity is very low, the lateral sizes in the structured melt are still larger. They are much greater than the critical nucleus size. After quenching to a lower temperature, they can grow spontaneously, without crossing the nucleation barrier. They can act as athermal nuclei, confirming previous conjecture. The methodologies proposed here help to better utilize synchrotron SAXS to study polymer crystallization.

## 5. Related literature   

The following references are cited in the supporting information for this article: Piccarolo *et al.* (1992) ▸.

## Supplementary Material

Supporting information. DOI: 10.1107/S2052252521003821/ed5023sup1.pdf
