# Supplementary material for: Detection and characterization of folded-chain clusters in the structured melt of isotactic polypropyl­ene
Source: IUCrJ. 2021 May 15;8(Pt 4):595–607. doi: 10.1107/S2052252521003821 (PMC8256699; doi:10.1107/S2052252521003821)
Supplement: Supplementary file 1 [file m-08-00595-sup1.pdf]

IUCrJ

Volume 8 (2021)

Supporting information for article:

Detection and characterization of folded-chain clusters in the structured melt of isotactic polypropylene

Xiangyang Li, Jianjun Ding, Pujing Chen, Kang Zheng, Xian Zhang and Xingyou Tian

S1. Scattering from Single Crystal Plane

Scattering from a crystal plane at q = 1 μm^{-1} was determined using the equation proposed by us earlier (Li et al. *IUCrJ* **2019,** *6*, 968–983):

$$I_\phi = I_e\left(\sum_{n=0}^{N-1}\cos n\phi\right)^2 = \left(\frac{\sin\frac{N\phi}{2}\cos\frac{(N+1)\phi}{2}}{\sin\frac{\phi}{2}}\right)^2 I_e \qquad (1)$$

where *N* is electron number, I_e is scattering intensity of single electron. ϕ is the phase difference between adjacent electrons, which is determined by following equation:

$$\phi = \ qa\sin(\theta_i - \theta) \qquad (2)$$

where q is wave vector, θ_i is incident angle, θ is scattered angle and a is the distance between adjacent electrons, as shown in Figure S1.

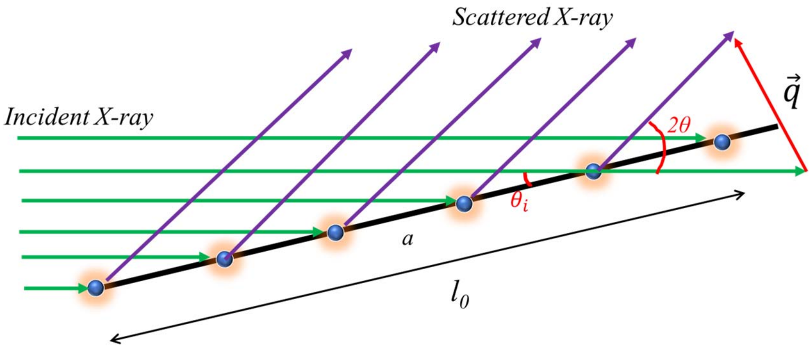

Figure S1 Scattering from single crystal plane

Same parameters employed in Figure 1a-b were used to determine the scattering at q = 1 μm^{-1}. I_e was assumed to be 1, a was assumed to be 0.17 nm, *N* was assumed to be 1471. The value of a is estimated from α crystal cell of iPP. The value of *N* is estimated from typical lateral size. Assuming the lateral size being 250 nm, the electron number *N* will be $N = \frac{l_0}{a} = 1471$.

With these parameters and the wave vector q = 1 μm^{-1}, scattering was determined employing Eq. 1and 2.

S2. Interfacial scattering induced by evanescent wave in SALS

Interfacial scattering induced by evanescent wave in SALS was determined with the equation proposed by us earlier (Li et al. *IUCrJ* **2019,** *6*, 968–983):

$$I_{\mathrm{ev}}^{\mathrm{i}}(q) = N_c{}^2 I_e e^{-\left(\frac{\lambda l_0 q^2}{8\pi}\right)^2} \left\{ \sum_{m=0}^{n\text{-}1} \left[\mathrm{e}^{\frac{-mL}{d_p^*}} \cos mLq - \mathrm{e}^{\frac{-(mL+d)}{d_p^*}} \cos\left(mL+d\right)q \right] \right\}^2 \quad (3)$$

where N_c is the electron number on the (*00l*) crystal plane, l_0 is lateral size of a lamellar stack, λ is the wave length employed, L is the long period of lamellar stack, d is lamellar thickness, and n is the number of lamellar crystals in the lamellar stack. It is from interference of every interfaces, as shown in Figure S2.

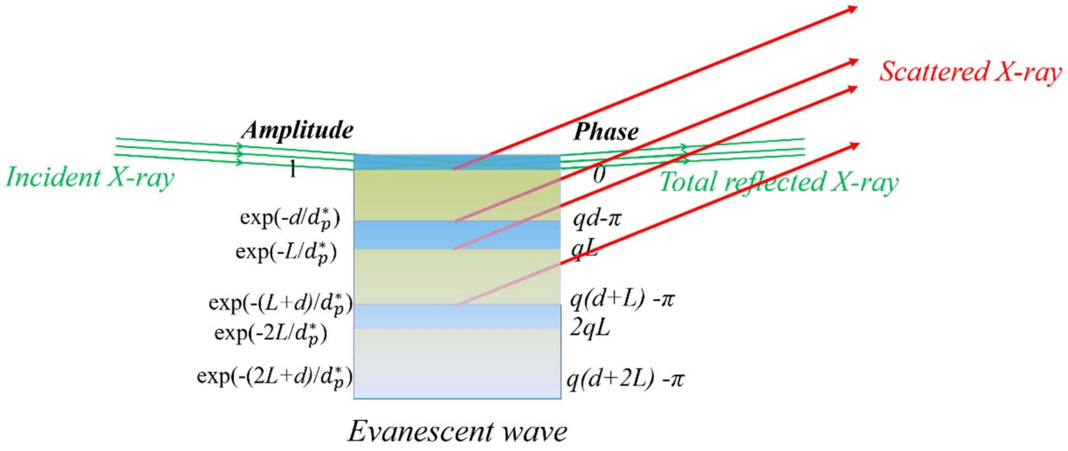

Figure S2 Scattering of interfacial electrons involved in the evanescent wave in a lamellar stack.

Same as previous study, N_c was assumed to be 1471, l_0 was assumed to be 250 nm, L was assumed to be 10.3 nm, d is assumed to be 7.3 nm and n is assumed to be 3. Different from previous study, λ was assumed to 632.8 nm, while the characteristic penetration depth d_p^* was assumed to be 1 μm, since d_p^* normally has a few wavelengths. Figure S3 shows interfacial scattering in SALS determined using above equation and parameters. For comparison, the scattering in SAXS is also given. It can be found that interfacial involved in the evanescent wave can induce strong scattering in SAXS but not in SALS, due to large d_p^*.

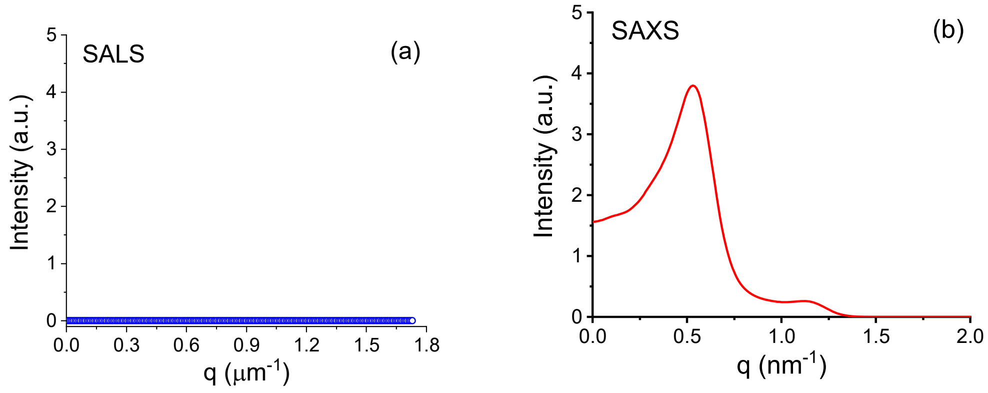

Figure S3 Interfacial scattering induced by evanescent wave in SALS (a) and SAXS (b).

S3. The influence of I_{ev}^{b}

The scattering of the bulk electrons I_{ev}^{b} can be determined with following equation:

$$I_{ev}^{b} = N_c^{\,2} I_e e^{-\left(\frac{\lambda l_0 q^2}{8\pi}\right)^2} \left\{\frac{q d_p^{*2}}{1+q^2 d_p^{*2}}\right\}^2$$

$$\left\{\frac{1}{q d_p^{*}} + \frac{\rho_c-\rho_a}{\rho_c}\left[\sum_{m=0}^{n-1} e^{-\frac{mL+d}{d_p^{*}}}\left(\sin q(mL+d) - \frac{\cos q(mL+d)}{q d_p^{*}}\right) - \sum_{m=1}^{n-1} e^{-\frac{mL}{d_p^{*}}}\left(\sin mqL - \frac{\cos mqL}{q d_p^{*}}\right)\right]\right\}^2 \quad (7)$$

where d is the lamellar thickness, L is long period, n is the number of lamellar crystals in the lamellar stack, d_p^* is the characteristic penetration depth of evanescent wave, and $\frac{\rho_c-\rho_a}{\rho_c}$ is the relative electron density difference between amorphous and crystalline layers. It is mainly from two parts:

$$I_{ev}^{decay} = N_c^{\,2} I_e e^{-\left(\frac{\lambda l_0 q^2}{8\pi}\right)^2} \left\{\frac{d_p^{*}}{1+q^2 d_p^{*2}}\right\}^2 \quad (8)$$

$$I_{ev}^{contrast} = N_c^{\,2} I_e e^{-\left(\frac{\lambda l_0 q^2}{8\pi}\right)^2} \left\{\frac{q d_p^{*2}}{1+q^2 d_p^{*2}}\frac{\rho_c-\rho_a}{\rho_c}\right\}^2 \quad (9)$$

$$\left[\sum_{m=0}^{n-1} e^{-\frac{mL+d}{d_p^{*}}}\left(\sin q(mL+d) - \frac{\cos q(mL+d)}{q d_p^{*}}\right) - \sum_{m=1}^{n-1} e^{-\frac{mL}{d_p^{*}}}\left(\sin mqL - \frac{\cos mqL}{q d_p^{*}}\right)\right]^2$$

The first part I_{ev}^{Decay} is from the decay of evanescent wave, while the second part $I_{ev}^{contrast}$ is from electron density contrast between amorphous and crystalline layers. The relative electron density difference in polymers is small. For example, the relative electron density difference is only 8% (Piccarolo et al. J. Appl. Polymer Sci. 1992, 46, 625–634.). Due to

weak electron density difference, the first part is much greater than the second part, as seen in Figure S4a.

At higher q, $qd_p^* \gg 1$. Eq. 8 can be reduced as:

$$\mathrm{I}_{ev}^{decay} \approx N_c^{\,2} I_e e^{-\left(\frac{\lambda l_0 q^2}{8\pi}\right)^2} \frac{1}{d_p^{*2} q^4} \qquad (10)$$

The scattering intensity is inversely proportional to q^4.

Assuming $N_c = 1471$, $I_e = 1$, $\lambda = 0.124$ nm, $l_0 = 250$ nm, $n = 3$, $d = 7.3$ nm, $L = 10.4$ nm $\frac{\rho_c - \rho_a}{\rho_c} = 0.08$ and d_p^*=6 nm, I_{ev}^i and I_{ev}^b are determined and plotted in Figure S4. Form Figure S4b, it can be found that scattering in I_{ev}^b concentrates mainly in small q range. It decreases monotonously. While scattering in I_{ev}^i distributes mainly in high q range. The long period peak is from I_{ev}^i.

In small q range, the scattering induced by evanescent wave I_{ev}^i is much greater than I_{ev}^b. This could be due to decay of evanescent wave in I_{ev}^b. As seen in Eq. 10, scattering intensity increases sharply with the decrease of q. Multiplying q^4 can suppress the scattering. As seen in Figure S4c, after multiplying q^4, I_{ev}^b becomes a small amount in the plot of Iq^4. Scattering induced by evanescent wave is controlled by I_{ev}^i.

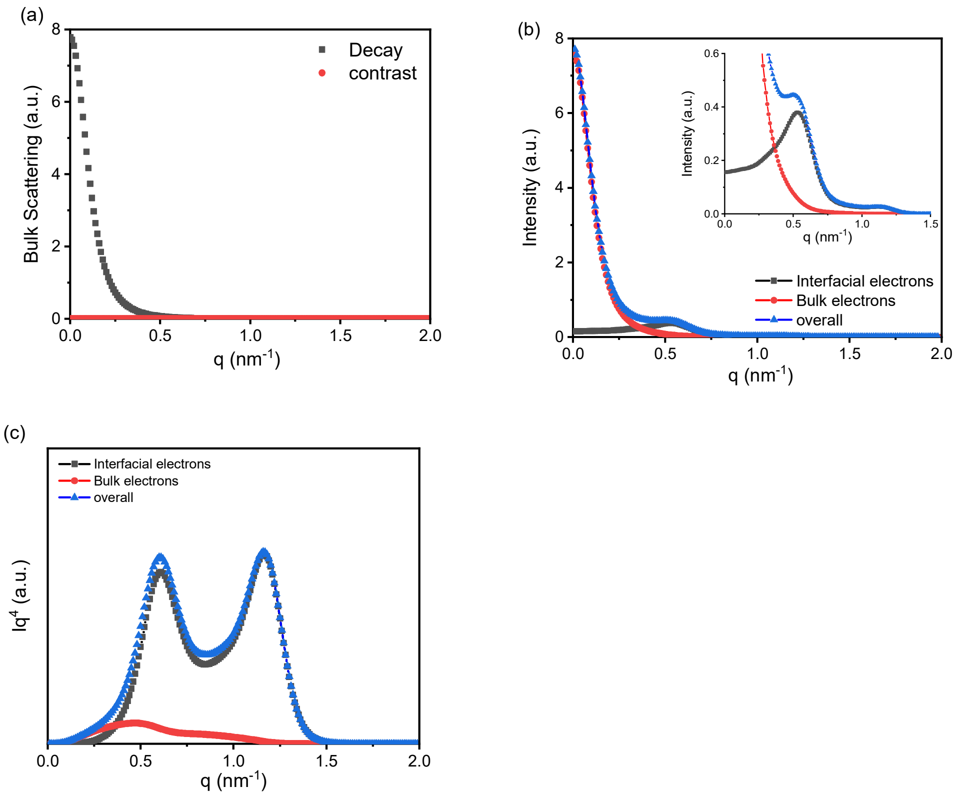

Figure S4 (a) Scattering due to the decay of evanescent wave I_{ev}^{Decay} and scattering due to electron density contract $I_{ev}^{contrast}$ in the scattering of the bulk electrons in a lamellar stack induced by evanescent wave. (b) I_{ev}^{i}, I_{ev}^{b} and overall scattering induced by evanescent wave determined by Eq. 3-5, where N_c = 1471, I_e = 1, λ = 0.124 nm, l_0 = 250 nm, n = 3, d = 7.3 nm, L = 10.4 nm and d_p^*=6 nm. (c) I_{ev}^{i} and I_{ev}^{b1} after multiplying q^4.
